# Radiologic stewardship for pediatric intracranial infections: opportunities to reduce low-yield follow-up MRI

**DOI:** 10.1017/ash.2026.10858

**Published:** 2026-10-06

**Authors:** Lydia Lu, Allyson Dalby, Adam Goldman-Yassen, Jacob Lepard, Preeti Jaggi

**Affiliations:** 1 Pediatric Infectious Disease, Children’s Healthcare of Atlanta Inc, Atlanta, USA; 2 Children’s Healthcare of Atlanta Inc, USA; 3 Emory University School of Medicine, USA; 4 Neurosurgery, Emory University School of Medicine, USA

## Abstract

Among 37 previously healthy children diagnosed with focal intracranial infections, 40 routine surveillance MRIs obtained >7 days postintervention in the absence of clinical symptoms did not alter surgical or medical management. Symptom-driven repeat imaging may safely replace routine protocols, reducing unnecessary sedation, contrast exposure, and environmental impact.

## Background

Diagnostic stewardship is a proven strategy to improve patient care through reducing inconsequential testing without sacrificing patient outcomes.^1^ Although prior efforts have focused on laboratory testing, radiologic diagnostics also affect patient management.^2^ Focal intracranial infections are rare but life-threatening complications of sinusitis, otitis media, and mastoiditis in previously healthy children.^3^ In addition to surgery and antimicrobial therapy, management of these infections has conventionally included repeat brain imaging to assess treatment response, however there are no evidence-based guidelines for this practice, or for interpreting residual radiologic findings in clinically improving patients.^4^ Unnecessary imaging poses direct patient and family harms, including medical anxiety, sedation risk, contrast exposure, procedural costs, and logistic burdens. Environmental costs are also substantial: MRI is the most energy-intensive imaging modality, accounting for nearly half of a radiology department’s greenhouse gas emissions (∼17.5 kg CO_2_e per scan), and gadolinium-based contrast agents raise concerns for persistent environmental contamination of water sources.^5–7^

Efforts to limit unnecessary brain imaging (CT and MRI) have been undertaken in children after trauma and headaches, however the utility of follow-up imaging for intracranial infections has not been addressed.^8^ We audited current practices at our institution, suspecting many follow-up studies added little to clinical management.

## Methods

This study was approved as a quality review by the Children’s Healthcare of Atlanta (CHOA) Institutional Review Board. CHOA is comprised of three free-standing children’s hospitals, two of which are staffed by neurosurgery, otolaryngology, and pediatric infectious disease (ID) subspecialists. We conducted a manual retrospective chart review of all previously healthy patients followed in ID clinic after hospitalization for subdural empyema, epidural empyema, or brain abscess attributed to extension from a paranasal sinus, middle ear or mastoid infection from Jan 2022—August 2024.

Patient demographics, diagnoses, surgical interventions, antibiotics and brain imaging were extracted. Order indications and provider notes surrounding each imaging study were reviewed, as were surgical interventions in the 14 days following an intracranial MRI. We documented subsequent management escalation (additional surgical procedures, broadened or prolonged antibiotics) and de-escalation (narrowed antibiotics or shortened courses). Images obtained within 7 days of diagnosis or surgical intervention, or those prompted by concerning clinical changes were identified as “symptomatic.” Images obtained >7 days from diagnosis or surgery without documented clinical symptoms were identified as “surveillance.” Head CT and MRI with venography were documented but excluded from this analysis due to their broader indications. Treatment complications of antibiotic therapy were similarly documented.

Descriptive statistics were calculated in Microsoft Excel for the overall population and for subgroups of those who had repeat MRI for management of intracranial infection.

## Results

### Demographic overview

We identified 37 patients followed from Jan 2022—August 2024 with subdural empyema (n = 17, 46.0%), epidural empyema (n = 13, 35.1%) and intraparenchymal brain abscess with or without epidural/subdural abscess (n = 7, 18.9%). Patients were mostly male (n = 27, 73.0%), Black or African American (n = 28, 75.7%), and enrolled in public health insurance (n = 20, 54.1%), with a median age of 12 (IQR 8–14 yr, range: 1–18). Antibiotic management and associated adverse events are also reported in Table 1.

**Table 1.** Patient demographics and clinical characteristics Table 1 long description.

Characteristic	Total sample N = 37
	n (%)
Sex	
Female	10 (27.0)
Male	27 (73.0)
Race	
Asian	1 (2.7)
Black	28 (75.7)
White	8 (21.6)
Age^a^	
< 12 years	14 (37.8)
≥ 12 years	23 (62.1)
Insurance type	
Public	20 (54.1)
Private	12 (32.4)
Not insured	5 (13.5)
Admitting service	
General pediatrics	6 (16.2)
Pediatric intensive care unit	31 (83.8)
Diagnosis	
Intraparenchymal brain abscess	7 (18.9)
Epidural empyema	13 (35.1)
Subdural empyema	17 (46.0)
Length of hospitalization^a^	
<13 days	18 (48.6)
≥ 13 days	19 (51.4)
Most invasive surgical procedure	
FESS^b^/Other^c^	11 (29.7)
Burr hole/Craniotomy	17 (46.0)
Craniectomy	8 (21.6)
No procedure	1 (2.7)
Number of surgical procedures	
0	1 (2.7)
1	18 (48.6)
2	17 (46.0)
3	1 (2.7)
Duration of antibiotics^b^	
<44 days	16 (43.2)
≥44 days	21 (56.8)
Emergency department visit postdischarge	
Yes	14 (37.8)
No	23 (62.2)
PICC^d^ Line complication	
Yes	5 (13.5)
No	32 (86.5)

a Median age of 12 years used to define groups, median length of hospitalization of 13 days, and median duration of antibiotics of 44 days used to define groups.

b Functional Endoscopic Sinus Surgery.

c Fenestration of cyst.

d Peripherally inserted central catheter.

### Neurosurgical interventions

Nearly all patients (n = 36, 97.3%) underwent neurosurgical intervention. Of these, 18 (50.0%) required more than one surgical intervention. The median time between the first and second neurosurgical procedures was 5 days (IQR 4–8.8 d).

### Cranial Imaging (excludes MRI and CT venogram)

Most patients (n = 24, 64.9%) were diagnosed by MRI at our institution; 11 patients by CT and 2 were transferred after outside diagnostic imaging. In total for our 37 patients, 86 MRIs were performed at our institution, averaging 2.3 MRIs (1.7 repeat) per patient throughout treatment.

#### Overall repeat MRI utilization

Most of our cohort had repeat MRI, a total of 62 studies across 31 patients. Of these, 20 (54.1%) were re-imaged during hospitalization, 20 (54.1%) after discharge. Fifteen patients (40.5%) underwent repeat MRI during both hospitalization and after discharge. Only 6 patients in the cohort (16.2%) did not have any repeat MRI.

#### Repeat MRI timing

Most patients (n = 21, 57%) in our cohort had at least one repeat MRI within 7 days (median 3, IQR 0–7 d) of first surgical intervention. The majority (n = 14, 67%) of these studies were triggered by clinical symptoms: most commonly fever, neurologic symptoms (headache, seizure), failure to improve, suspected surgical site infections, or elevated inflammatory markers. Among symptomatic children, 8 (57.0%) required a second surgical intervention after repeat MRI. For the remaining studies not associated with symptoms, clinicians documented that repeat imaging was indicated to confirm the success of surgical drainage, but none of these studies led to management escalation.

**Figure 1.**
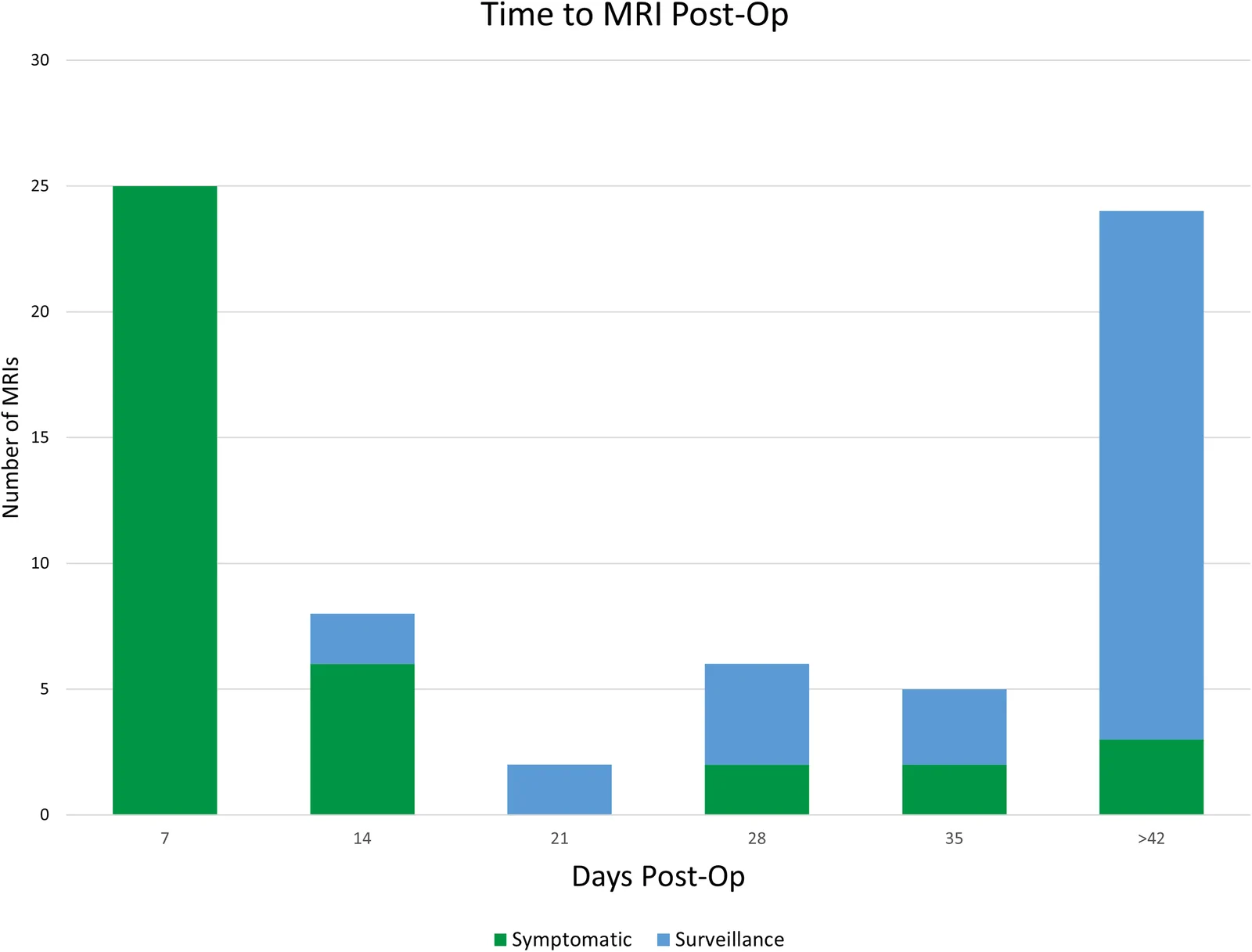
Time to postoperative MRI. Notes: Time to second procedure (n = 18 patients): Median 5 days (IQR: 4–8.8 d). Symptomatic: Images obtained within 7 days of diagnosis or surgical intervention, or those prompted by concerning clinical changes. Surveillance: Images obtained after 7 days of diagnosis or surgical intervention and without concerning clinical changes.

An additional 40 MRI (64.5% of repeat studies) were obtained for routine surveillance, without documented clinical symptoms from 26 patients >7 days from diagnosis or surgery, at a median 37 days (8–166). (Figure 1) No repeat MRI >7 days from diagnosis or surgery led to management escalation or de-escalation.

#### Need for sedation

Ten (27.0%) patients required sedation to obtain repeat imaging for a total of 14 MRI studies. Two children required sedation three times each, and one child underwent general anesthesia. In all, 5 (35.7%) of the repeat MRI under sedation were obtained >7 days from diagnosis or surgery.

## Discussion

In our cohort, the first week following surgical intervention was the most critical period for imaging reassessment but repeating MRI imaging >7 days from surgical intervention without concerning symptoms did not lead to a change in management. These findings are consistent with the European Society of Clinical Microbiology and Infectious Diseases (ESCMID) brain abscess guidelines, which recommend immediate imaging for clinical deterioration and suggest in a good clinical practice statement interval imaging “until clinical cure is evident,” but do not define whether imaging is necessary in clinically improving patients. ESCMID further cautions that it is “often inappropriate to prolong antimicrobial treatment based solely on” residual radiologic findings, a principle supported by the absence of management escalation from surveillance imaging in our cohort.^1,9^

Our cohort underwent 40 MRI for surveillance purposes; none led to management escalation. As a radiologic stewardship target, unnecessary MRI are analogous to unnecessary blood cultures.^1^ Eliminating these 40 MRI would have avoided about 700 kg CO_2_e (equivalent to burning 778 pounds of coal), associated gadolinium release into wastewater and 5 sedation events, with no impact on patient outcomes.

We conducted a single-center, hypothesis-generating study with a small sample size, limiting the strength and generalizability of our assertions. Our methodology did not capture patients who completed treatment during prolonged hospitalizations or were lost to follow-up. Retrospective design also limited us to available documentation which may not fully capture clinical decision-making. Multicenter prospective studies are needed to further test the 7-day symptoms threshold we noted as a starting framework. Future work should incorporate cost-effectiveness analyses and quantify burdens to patients and families, including those from CT scans with associated radiation exposure. Additionally, as current ESCMID recommendations address brain abscess specifically, their applicability to subdural and epidural empyema, most of our cohort, has not been established and the scope of future guidelines should be broadened to include these indications.

In conclusion, we identified opportunities to steward diagnostic imaging for previously healthy children with intracranial infections consistent with joint position statements addressing radiologic sustainability.^10^ Extending diagnostic stewardship principles to MRI can benefit patients and systems by reducing risks of sedation, contrast exposure and environmental costs without adverse outcomes.

## Table 1. Long description

A table detailing patient demographics and clinical characteristics for 37 patients. The table has 15 rows and 2 columns. The first column lists characteristics such as sex, race, age, insurance type, admitting service, diagnosis, length of hospitalization, most invasive surgical procedure, number of surgical procedures, duration of antibiotics, emergency department visit postdischarge, and PICC line complication. The second column provides the total sample size and percentage for each characteristic. For example, 27.0 percent of patients are female, 73.0 percent are male, 2.7 percent are Asian, 75.7 percent are Black, 21.6 percent are White, 37.8 percent are under 12 years old, 62.1 percent are 12 years or older, 54.1 percent have public insurance, 32.4 percent have private insurance, 13.5 percent are not insured, 16.2 percent are admitted to general pediatrics, 83.8 percent are admitted to the pediatric intensive care unit, 18.9 percent have intraparenchymal brain abscess, 35.1 percent have epidural empyema, 46.0 percent have subdural empyema, 48.6 percent have a hospitalization length of less than 13 days, 51.4 percent have a hospitalization length of 13 days or more, 29.7 percent undergo FESS/Other procedures, 46.0 percent undergo Burr hole/Craniotomy, 21.6 percent undergo Craniectomy, 2.7 percent have no procedure, 2.7 percent have zero surgical procedures, 48.6 percent have one surgical procedure, 46.0 percent have two surgical procedures, 2.7 percent have three surgical procedures, 43.2 percent have antibiotics for less than 44 days, 56.8 percent have antibiotics for 44 days or more, 37.8 percent visit the emergency department postdischarge, 62.2 percent do not, 13.5 percent have PICC line complications, and 86.5 percent do not.

Navigate back to Table 1..
